# Cheiro-Oral syndrome caused by thalamus hemorrhage: A case report

**DOI:** 10.1097/MD.0000000000009652

**Published:** 2018-01-12

**Authors:** Yen-Hung Wu, Kuan-Ting Liu, I-Jeng Yeh, Chia-Wen Chang

**Affiliations:** a Department of Emergency Medicine, Kaohsiung Medical University Hospital; b School of Medicine, College of Medicine, Kaohsiung Medical University, Kaohsiung, Taiwan.

**Keywords:** Cheiro-Oral syndrome, hemorrhagic stroke, thalamus

## Abstract

**Rationale:**

Cheiro-Oral syndrome (COS) is a pure sensory deficit confined to the perioral area and ipsilateral distal fingers or hand. Owing to relatively minor clinical findings and various presentations in different cases, the insidious and severe illness it implies may be overlooked at acute settings.

**Patient concerns:**

A 70-year-old man with history of hypertension and type II diabetes mellitus under regular medication control came to our emergency department with chief complaint of sudden onset of right perioral region and right upper limb numbness. General physical and neurological examinations were normal except for subtle hypoesthesia to light touch, and pinprick in the right corner of mouth and right forearm to distal fingers.

**Diagnoses:**

Routine blood analysis was all in normal range including white blood cell count, hemocrit platelet, renal and liver function, and electrolytes such as sodium and potassium. Noncontrast brain computed tomography showed abnormal high-attenuation collection in the left thalamus.

**Intervention:**

Follow-up computed tomography showed absorption of the hemorrhage after strict control of his blood pressure.

**Outcomes:**

The patient was discharged 7 days later from our hospital with stable condition.

**Lessons:**

We demonstrated type I COS associated with thalamic hemorrhage to highlight the neurological implication of COS. It is crucial for emergency clinicians to recognize the symptoms and promptly order a neuroimaging study to exclude large infarction/hemorrhage, which would deeply affect the disposition and following treatment of the patient.

## 1 Introduction

Cheiro-Oral syndrome (COS) is a pure sensory deficit confined to the perioral area and ipsilateral distal fingers or hand. Because of to relatively minor clinical findings and various presentations in different cases, the insidious and severe illness it implies may be overlooked at acute settings. Here, we report a patient with the diagnosis of left thalamic hemorrhage whose clinical manifestation was compatible with COS. After we contacted the regulations of institutional review board of the Kaohsiung Medical University Hospital, there was no need for ethical approval for this case report article.

## 2 Case present

**Figure 1:**
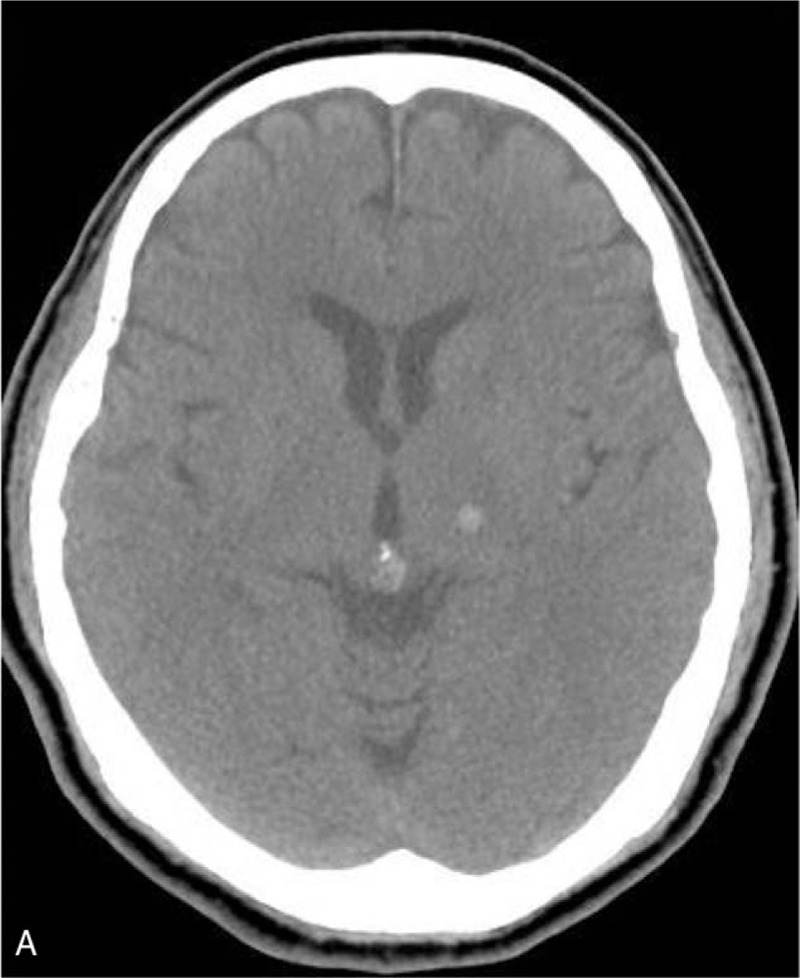
Computed tomography showing left thalamus hemorrhage (axial view).

**Figure 2:**
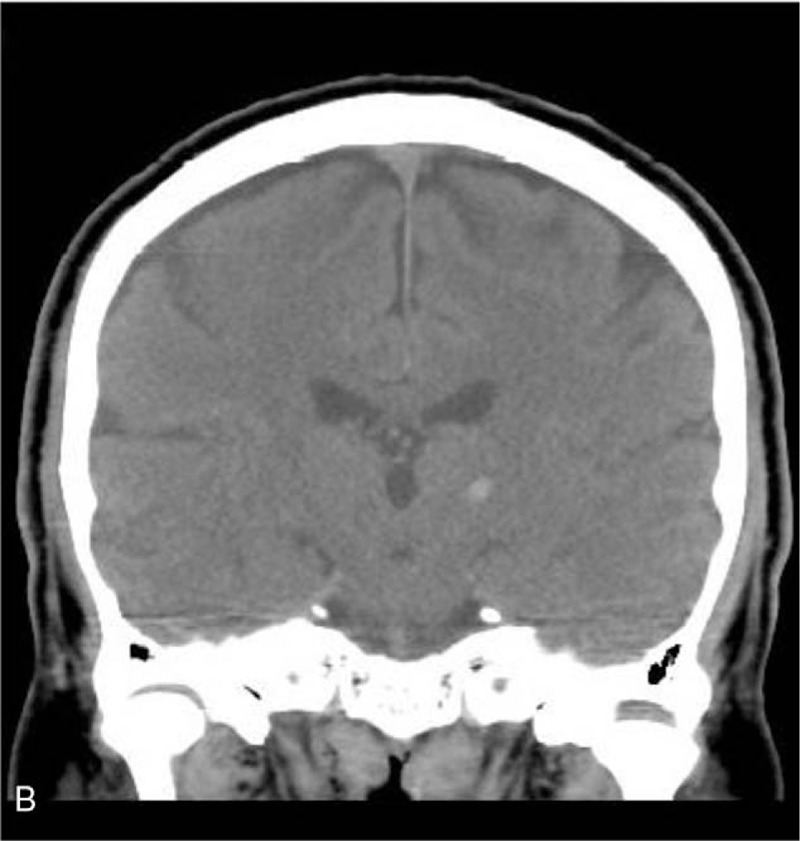
Computed tomography showing left thalamus hemorrhage (coronal view).

A 70-year-old man with history of hypertension and type II diabetes mellitus under regular medication control came to our emergency department with chief complaint of sudden onset of right perioral region and right upper limb numbness. On arrival to our emergency department, he was clear and alert. Blood pressure was 197/88 mmHg. General physical and neurological examinations were normal except for subtle hypoesthesia to light touch, and pinprick in the right corner of mouth and right forearm to distal fingers. No other cranial nerve deficits or pathological reflexes were observed. Routine blood analysis was all within normal range including white blood cell, hemocrit, platelet, renal and liver function, and electrolytes such as sodium and potassium. However, noncontrast brain computed tomography showed abnormal high-attenuation collection in the left thalamus (Figs. 1 and 2). Under the diagnosis of left thalamic hemorrhage, this patient was admitted to neurosurgery intensive care unit for further observation. Follow-up computed tomography showed absorption of the hemorrhage after strict control of his blood pressure. The patient was discharged 7 days later from our hospital with stable condition.

## 3 Discussion

COS is reported in the literature as having a number of variants based on the distribution of sensory impairments. Four types of COS are categorized. Type I COS is sensory impairment confined to perioral area and homolateral finger(s)/hand in unilaterality. Type II COS is sensory impairment confined to perioral area and finger(s)/hand in bilaterality. Type III COS is sensory impairment confined to perioral area and finger(s)/hand in that one is involved bilaterally whereas another one is unilateral. Type IV COS is sensory impairment confined to the perioral area and opposite finger(s)/hand, or crossed COS. Type I COS accounts for the largest number of people (71.1%) and type IV accounts for the fewest (6.5%).^[1]^ In this case, the patient was a type I COS because of the sensory impairment confined to the right perioral and right hand /fingers.

Corresponding etiologies identified include ischemic^[2]^ or hemorrhagic^[3]^ stroke, tumor or vascular malformation,^[4]^ and encephalitis. Among them, small infarction is the leading cause.^[1]^ Hemorrhagic stroke was the cause of COS in this patient.

The location of the lesion in most cases arises from the pons, thalamus, cortex, and medullary oblongata. The spine may be the location of another possible unusual lesion seen in previous literature.^[5]^ Type IV COS is also called crossed COS and is predictive for medullary involvement, ischemia, and neurological deterioration.^[6]^ Cortical involvement can also predict deterioration.^[1]^

## 4 Conclusion

In our case, we demonstrate typed I COS associated with thalamic hemorrhage to highlight the neurological implication of COS. It is crucial for emergency clinicians to recognize the symptoms and promptly order a neuroimaging study to exclude large infarction/hemorrhage, which would deeply affect the disposition and following treatment of the patient.
